# Deep vein thrombosis following the treatment of lower limb pathologic bone fractures – a comparative study

**DOI:** 10.1186/s12891-018-2141-4

**Published:** 2018-07-11

**Authors:** Mihail-Lazar Mioc, Radu Prejbeanu, Dinu Vermesan, Horia Haragus, Marius Niculescu, Daniel Laurentiu Pop, Andrei Dan Balanescu, Daniel Malita, Bogdan Deleanu

**Affiliations:** 1 Timisoara Emergency Clinical County Hospital, Liviu Rebreanu Bvd, No 156, 300723 Timisoara, Romania; 2 “Victor Babes” University of Medicine and Pharmacy, Eftimie Murgu Square, No 2, 300041 Timisoara, Romania; 3 “Titu Maiorescu” University, Dambovnicului Str, No 22, District 4, Bucharest, Romania

**Keywords:** Deep vein thrombosis, Pathologic bone fracture, Musculoskeletal tumour, Anticoagulation protocol

## Abstract

**Background:**

Deep vein thrombosis is a well-known complication of fracture occurrence, lower limb surgery and periods of prolonged immobilisation. Its incidence can be increased even more in specific cases with metastatic bone disease and adjuvant treatment. There is a small amount of literature that addresses the incidence of DVT by comparing osteosynthesis and arthroplasty as surgical treatments. Current recommended anticoagulation protocols might be inadequate for specific groups of cancer patients undergoing osteosynthesis or arthroplasty.

**Methods:**

The study was designed and performed in a retrospective manner and carried out on patients that presented at our Emergency Clinical County Hospital between 01.01.2008–31.12.2016. The patients’ evolution was followed for a standard of 2 months. All our deep vein thrombosis events were diagnosed via venous duplex imaging. The studied lot (*n* = 85) was paired with a control group (*n* = 170) with similar baseline characteristics.

**Results:**

Our lot was comprised of 85 patients that underwent 85 surgeries, on both of our hospital’s Orthopaedic and Traumatology wards. When performing the student t-test and calculating OR (odds ratio) and RR (risk ratio) we encountered 11 cases of DVT in our studied group and 12 cases of DVT in our control group (*p* < 0.04). We found statistical significance when correlating DVT with type of implant (prosthesis), the presence of metastases over primary tumour and the choice of implant (prosthesis over intramedullary nail). There was no statistical significance found when correlating DVT events with the type of anticoagulation and the amount of blood transfusion units required.

**Conclusion:**

Patients who undergo surgical treatment for lower limb pathological fracture due to malignancy are at increased risk of DVT or death due to PE under current general thromboprophylaxis regimens. The risk is higher for the immediate postoperative period (10 days). The risk is increased by metastasis, arthroplasty and adjuvant therapy (radiotherapy, chemotherapy), and we think that a more aggressive prophylactic protocol should be used.

## Background

Pathologic bone fractures (PBO) are one of the most feared types of pathologic bone events, resulting in an increased state of disability to a group of patients that already has a certain degree of social dependence, and poses a great vital risk. This vital risk is represented by the specific fracture complications, the treatment (surgical, conservative) of the fracture and the association between the fracture and the neoplastic disease. As the economic effort regarding the treatment of patients with metastatic bone disease began to grow, it was then shown that it is more cost-effective to perform prophylactic surgery than to treat pathologic bone fractures [1]. Recent reports show that both osteosynthesis rates and life expectancy in patients with metastatic bone disease are growing [2] and due to that so are the complication rates. Even though more and more authors are pushing for even further enhanced functional results following lower limb fractures [3], one must consider the sometimes, lethal complications that may occur.

Deep vein thrombosis (DVT) is a well-known complication of fracture occurrence, lower limb surgery and periods of prolonged immobilisation. The first description of the main risk factors for developing VTE were described by Virchow in 1846, and Virchow’s triad as it is known today consists of blood flow alterations, vascular endothelial injury and the hypercoagulable state [4]. Even with prophylactic treatment, the risk of orthopaedic treated patients (hip and knee arthroplasty) to develop a thrombotic event is estimated somewhere between 0.5–1% [5]. Cancer-related hypercoagulability is a well-known issue. It is predominantly caused by one of the following: tumour invasion, vascular compression, radiotherapy and some types of chemotherapy [6]. The incidence of venous thromboembolic events (VTE) in cancer patients is reported at 11% [7], while the surgical orthopaedic subgroup is at an even higher risk [8]. The greatest therapeutic challenge remains in obtaining a balance with the antithrombotic treatment as to avoid deep vein thrombosis and severe postoperative bleeding or wound complications [9]. Computer assisted interventions are becoming an integrated part of medicine in the hopes of better outcomes and faster recovery times [10]. Lately, advanced imaging solutions such as the computer tomography and the magnetic resonance imaging, have allowed us to diagnose several types of affections and complications related to the skeletal system [11].

Lately, there has been a great deal of interest in this field of research due to its high significance in morbidity and mortality. Regardless, there is a small amount of literature that addresses the incidence of DVT by comparing osteosynthesis and arthroplasty as surgical treatments. Current recommended anticoagulation protocols might be inadequate for specific groups of cancer patients undergoing osteosynthesis or arthroplasty.

## Methods

The study was designed and performed in a retrospective manner and carried out on patients that presented at our Emergency Clinical County Hospital between 01.01.2008–31.12.2016. We searched for our patients in our electronic database by imputing the ICD-10 codes that represented lower limb fractures – S72.x, S82.1–3 (femur and tibia), malignant bone lesions – C40.x, C41.x, D49 and we built our studied lot by associating these codes, thus resulting with the number of patients that had a pathologic bone fracture.

Our lot was comprised of 85 patients that underwent 85 surgeries, on both of our hospital’s Orthopaedic and Traumatology wards. Our studied lot was divided into two subgroups – patients receiving osteosynthesis and patients receiving arthroplasty. We matched our 85-patient lot with a control group (170 patients) of similar age, gender ratio and received surgical treatment with lower limb fractures without any kind of bone malignancy diagnosis. These patients have been treated at the same hospital, in the same timeframe and by the same surgeons. This lot’s baseline characteristics were also extracted through chart analysis.

We recorded the type of malignancy (primary, secondary) and its histologic origin. We noted any suspicion of DVT (leg pain and tenderness, erythema, oedema) and if it was confirmed on venous duplex imaging during admission. The venous duplex was performed by a highly skilled (over 80 duplexes for DVT/year) radiologist from the radiology section. We took notice of the type of antithrombotic treatment that the patients received while being admitted in the hospital. Known preoperative radiotherapy and postoperative chemotherapy was also taken into consideration, as they may influence the risk for DVT occurrence. The type of used implants was recorded together with the location of the fracture and the amount of perioperative and postoperative blood transfusions that were needed.

Odd ratio and relative risks were established for DVT in relation with the existence of malignancy, the type of used implant, the usage of radio or chemotherapy, the thromboprophylaxis protocol and the amount of blood units received by the patient. Student’s t-test was used to compare patients from both groups when possible. We established that a *p* value under 0.04 represents statistical significance. For significant data, our confidence intervals were set at 95%. For our statistical analysis we used SPSS 20 (Copyright IBM Corporation 2011).

All procedures performed in studies involving human participants were in accordance with the ethical standards of the institutional research committee and with the 1964 Helsinki declaration and its later amendments or comparable ethical standards. This study was approved by our hospital’s Institutional Review Board. All our patients sign a written consent when they are admitted in the hospital by which they agree that their medical information can be used for scientific purposes without breaking confidentiality.

## Results

Our gender distribution was divided into 38 males and 47 females, while the mean age of our patients was 61.9 years old (27–85, SD = 9.31). Most of our patients were affected by metastatic bone disease (*n* = 55) and the rest had different primary malignant tumours (*n* = 30). The baseline characteristics of both our target and our control groups can be found in Table 1.

**Table 1:** Baseline characteristics of our target and control groups

	Study group (*n* = 85)		Control group (*n* = 170)	
	Non DVT	DVT	Non DVT	DVT
Number of patients	74 (87%)	11 (13%)	158 (92.9%)	12 (7.1%)
Age (mean)	27–85 (61.9)	57–84 (68.4)	25–85 (62.1)	32–81 (59.7)
Sex ratio				
Male/Female	33 (44.6%)/41 (55.4%)	5 (45.4%)/6 (54.6%)	71 (44.9%)/86 (55.1%)	5 (41.6%)/7 (58.4%)
Fracture location				
Femur	66 (89.1%)	11 (100%)	142 (89.8%)	11 (91.6%)
Tibia	8 (10.9%)	0	16 (10.2%)	1 (8.4%)
Type of implant				
Prosthesis	18 (24.3%)	2 (18.1%)	37 (23.4%)	4 (33.3%)
Intramedullary nail	53 (71.6%)	7 (63.8%)	112 (70.8%)	5 (41.6%)
Plate	3 (4.1%)	2 (18.1%)	9 (5.8%)	2 (16.7%)
Tumoral origin				
Metastases	47 (55.3%)	8 (72.7%)	0	0
Primary tumour	27 (44.7%)	3 (27.3%)	0	0
Adjuvant therapy				
Radiotherapy	18 (21.17%)	6 (54.5%)	0	0
Chemotherapy	15 (17.64%)	4 (36.3%)	0	0
None	41	1	158 (100%)	12 (100%)
Tromboprophylaxis				
LMWH	61 (82.7%)	10 (90.9%)	129 (81.7%)	9 (75%)
Double dose LMWH	4 (5.5%)	1 (9.1%)	9 (5.7%)	2 (16.6%)
Rivaroxaban	4 (5.5%)	0	6 (3.8%)	0
Clopidogrel	5 (5.9%)	0	14 (8.8%)	1 (8.4%)

### Baseline characteristics

Selected postoperative complications occurred as follows: wound necrosis (*n* = 6), excessive wound bleeding (*n* = 4), infection (*n* = 5). The thromboembolic prophylaxis was done with low molecular weight heparin (LMWH) (nadroparin – 42, enoxaparin – 33) in recommended prophylactic doses. Some patients have had pre-existent oral anticoagulation with rivaroxaban or clopidogrel, due to associated disease.

**Fig. 1:**
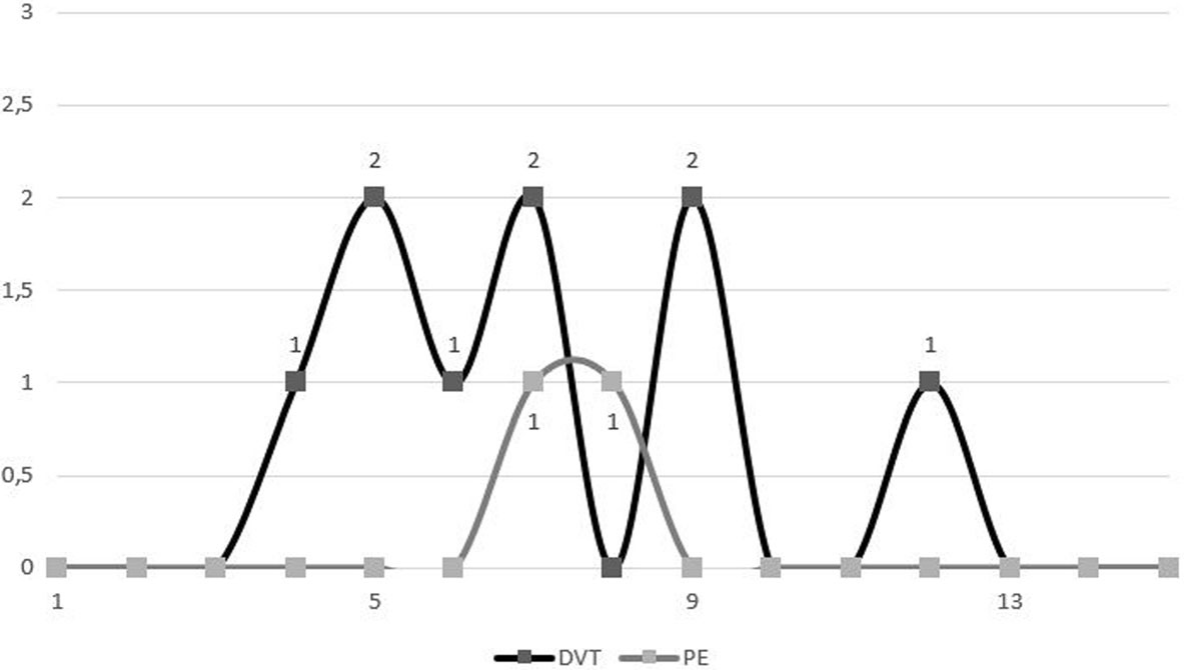
Graphical representation of the VTE events according to the day of occurrence

When performing the student t-test and calculating OR (odds ratio) and RR (risk ratio) we encountered 11 cases of DVT in our studied group and 12 cases of DVT in our control group (OR = 1.97, RR = 1.84, *p* < 0.04, CI = 95%). A significant percentage of the encountered thrombotic events occurred between the 5th and 10th postoperative days for the study group, as it can be seen in Fig. 1.

We noticed that it is highly unlikely for patients with intramedullary nails to develop DVT compared to patients with knee prostheses (OR = 0.11, RR = 1.16). We found statistical significance when correlating the DVT and PE events with the metastatic bone disease (*p* < 0.04, CI = 95% OR = 1.53, RR = 1.45). Immediate postoperative mortality caused by PE was only seen in metastatic disease (both pulmonary carcinoma). Adjuvant therapy in relation with DVT events proved significant (OR = 12.62, RR = 10.08). There was no statistical significance found when correlating DVT events with the type of anticoagulation (OR = 0.21, RR = 0.98) and the amount of blood transfusion units required (OR = 0.14, RR = 0.77).

## Discussion

Patients who undergo surgical treatment for lower limb pathological fracture due to malignancy are at increased risk of DVT or death due to PE under current general thromboprophylaxis regimens. The risk is higher for the immediate postoperative period (10 days). The risk is increased by metastases, arthroplasty and adjuvant therapy (radiotherapy, chemotherapy).

Regarding demographics, we observed a change in the peak age intervals, unlike Patrascu et al. reported in 2014. In a 5-year single centre retrospective study on musculoskeletal tumours they found that the graphical age distribution of their patients had 2 peaks (between decades 2–3 and 5–6) [12], whereas our patients were mostly (*n* = 68) over 50 years old. The considerable number of patients with metastatic bone disease was probably the cause of that, and somehow influenced the goal of our treatment that was often palliative.

The total wound complication rate was low, considering all the associated pathologies of our patients. Excessive wound bleeding is known to occur especially in arthroplasties, due to the approach and dissection required to perform the surgical intervention which is added to the anticoagulation treatment [13]. Some authors suggested that in such cases, postoperative blood loss can be countered by the administration of intraoperative and postoperative tranexamic acid [14]. No correlation could be made between TVP events and a certain type of thromboprophylaxis. Wound necrosis rates (7%) were not significantly higher than other surgical interventions performed on specific high-necrosis-risk areas. In our opinion, radiotherapy did not influence the outcome of the wound, even though there are a few studies suggesting this [15, 16].

Blood transfusions are known to increase the risk of VTE events in cancer patients. Xenos et al. analysed a lot of over 20,000 patients that had colorectal resection for cancer and concluded that the subset of patients that received intraoperative RBC transfusions were at an even higher risk for VTE [17]. They suggested a cautious use of blood transfusions during cancer resection. On an even bigger study, Khorana et al. found rates of 7.2 and 5.2% for cancer patients that received RBC transfusions to develop venous and arterial thromboembolism respectively. These rates were significantly higher than the control group (3.8 and 3.1%) [18]. Our results could not point out to a significant result, because many of our patients have had intraoperative or postoperative transfusions (69.15%). As acrylic cement spacers represent a viable surgical option for many tumours [19], and it is well known that cementing a hip prosthesis for example may have an impact on the patient’s hemodynamic [20, 21], we believe this could be something to look out for.

Regarding thromboprophylaxis, the majority of the VTE events occurred in patients treated with some sort of LMWH (81.81%). The American Society of Clinical Oncology, recommends that patients undergoing major surgery, should start LMWH prophylaxis before surgery, and that they continue it for 7–10 days. They also suggest LMWH as proper treatment for DVT and PE and secondary prophylaxis [22]. Oral anticoagulation is still a controversial treatment in orthopaedics and trauma and we believe that it is too early to properly determine its therapeutic efficacy.

The timing of DVTs was poorly assessed due to the study design and our hospital’s internal database. We believe that the postoperative period on which we had information regarding patient evolution was too short, and that if given a longer follow-up period the number of DVTs could grow. Bergqvist et al. stated that they found significant difference in the occurrence of postoperative VTE for 3 months, on a lot with patients that underwent abdominal cancer surgery [23]. The occurrence of VTE was statistically significant in correlation with the intramedullary nailing of femur fractures. Ratasvuori et al. performed a study that aimed to evaluate the risk of VTE in skeletal metastatic surgery. Pulmonary metastases and intramedullary nailing were 2 of the 3 independent risk factors for VTE [24]. Also, we think that having an even larger patient sample could further benefit our results regarding the power calculations and increase our manuscripts’ significance.

## Conclusion

Patients who undergo surgical treatment for lower limb pathological fracture due to malignancy are at increased risk of DVT or death due to PE under current general thromboprophylaxis regimens. The risk is higher for the immediate postoperative period (10 days). The risk is increased by metastasis, arthroplasty and adjuvant therapy (radiotherapy, chemotherapy), and we think that a more aggressive prophylactic protocol should be used.

## Abbreviations

CT: Computed tomography
DVT: Deep vein thrombosis
LMWH: Low molecular weight heparin
PBO: Pathologic bone fracture
PE: Pulmonary embolism
RBC: Red blood cells
VTE: Venous thromboembolism
